# Marsupialization of Dentigerous Cysts Followed by Enucleation and Extraction of Deeply Impacted Third Molars: A Report of Two Cases

**DOI:** 10.7759/cureus.23772

**Published:** 2022-04-02

**Authors:** Nedal Abu-Mostafa

**Affiliations:** 1 Oral and Maxillofacial Surgery and Diagnostic Sciences, Riyadh Elm University, Riyadh, SAU

**Keywords:** third molar, surgical extraction, marsupialization, enucleation, dentigerous cyst

## Abstract

A dentigerous cyst (DC) is a developmental odontogenic cyst that involves the crown or a portion of the crown of an unerupted or impacted tooth. Mandibular third molars are the most commonly implicated teeth in this type of cyst. DC can be asymptomatic and detected by an ordinary radiographic examination.

Two massive DC with deeply impacted third molars were treated by marsupialization followed by surgical extraction and enucleation of the residual lining. In both cases, sensation on the lower lip and chin remained normal, and complete bone healing was achieved.

Decompression by simple marsupialization and extended follow-up are important roles in bone deposition and reduction of the cyst. Furthermore, surgical extraction can be performed non-traumatically for the cyst-associated tooth, because the surrounding bone is newer and less calcified than mature bone.

## Introduction

Dentigerous cysts (DC) are the second most common odontogenic cysts after radicular cysts, accounting for 37.9% - 84.5% of all odontogenic cysts [1]. The crown or a portion of the crown of an unerupted or impacted tooth is surrounded by a DC. Mandibular third molars, maxillary permanent canines, mandibular premolars, and maxillary third molars are the most commonly implicated teeth, however, DC can also form with unerupted supernumerary teeth or odontomas [2].

DC is caused by an alteration of the reduced enamel epithelium (after completion of amelogenesis) that results in fluid accumulation between it and the enamel of the crown. The cyst lining has non-keratinized stratified squamous epithelium [3]. The progression of DC is slow and asymptomatic, and it can be detected through routine radiographic evaluation or investigation of failed tooth eruption or misalignment. When it becomes infected, though, pain may ensue. Long-term cysts can cause bone enlargement, resorption, and tooth displacement. It appears on radiograph films as a unilocular well-circumscribed radiolucency (greater than 5 mm) with a sclerotic border surrounding the crown of an unerupted tooth [4].

Enucleation or Marsupialization can be used to treat DC. Enucleation is a surgery that involves extracting the affected tooth and shelling out the entire cystic lining. The candidates for this treatment are small DCs that are away from vital structures, do not cause bone weakness, and involve supernumerary teeth or teeth that are not expected to erupt even with orthodontic extrusion [5]. 

The procedure of marsupialization can be performed under local anesthesia. A window is formed on the cystic wall, the cyst fluid is evacuated then the cystic lining should be sutured to the oral mucosa. These steps cause decompression that lowers intra-cystic pressure and encourages the bone formation and decreases the size of the cystic cavity. Marsupialization is the preferred therapy for large cysts adjacent to vital structures such as the maxillary sinus and inferior alveolar nerve. Other benefits of this method include facilitating cyst-associated tooth eruption and avoiding injury to adjacent anatomical features. However, the adverse circumstances are: the bulk of cystic lining is left in situ, the procedure necessitates numerous follow-up visits, cleaning difficulty, and the possibility of infection [6].

Marsupialization may not be able to cure the cystic cavity completely or stimulate the cyst-associated tooth to fully erupt. When adequate bone deposition has occurred to diminish jaw weakness and cover nearby important structures, it should be followed by enucleation at the proper time [7].

Two cases of deeply impacted third molars with massive DCs are described in this article. Marsupialization was used to treat them, followed by an atraumatic surgical extraction and enucleation of the residual lining. In both cases, the sensation on the lower lip and chin remained normal after the follow-up time. The treatment protocol resulted in full bone repair at the extraction sites with no subsequent complications.

The author was responsible for the entire surgical treatment and follow-up sessions in both cases. Informed consent was obtained from the two patients who had DCs. This case report was registered at the University's Research Center, and the Institute Review Board approved it with the registration number FRP/2021/376/592/566. The study complied with the World Medical Association’s Declaration of Helsinki.

## Case presentation

Case 1

In July 2018, a 25-year-old healthy male patient visited the clinics of Riyadh Elm University complaining of painless swelling on the right side of the lower jaw that started three months ago and increased in size gradually.

Clinical Examination

Extra-orally, there was facial asymmetry as well as a small bony hard enlargement on the mandible's right angle. The overlying skin was normal, with normal sensibility in the lower lip and no lymphadenopathy. An intra-oral examination revealed a missing lower right third molar and mild buccal cortical enlargement on the angle of the jaw, which was covered by normal mucosa.

Radiographic Examination

**Figure 1:**
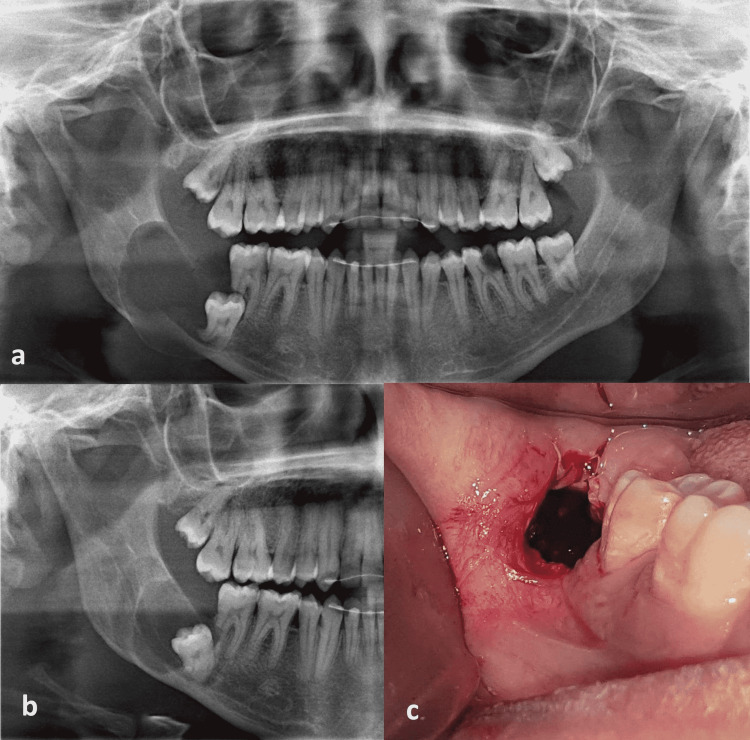
Initial panoramic radiograph (a) A large cystic lesion and the impacted 3rd molar (b) Six months after needle aspiration, the cystic cavity shrank (c) A window was created as a part of marsupialization.

A panoramic radiograph (Figure 1) showed a large unilocular radiolucent lesion enveloping the crown, distal and mesial roots of the lower right 3rd molar, with a well-defined radiopaque boundary. The lesion extended from the mandible's body at the lower 2nd molar area to the ramus's middle. It caused weakening and expansion of the buccal cortex around the mandible's lower border. The lower right third molar was deeply displaced near the lower border of the mandible, vertically angulated, had a distally curved root, and caused resorption on the distal root of the lower second molar (Figure 1a).

As a part of the clinical examination, a diagnostic needle aspiration was performed by a plastic disposable 3cc syringe with a 25 gauge needle the same day to check for cystic content. The fluid was a straw color and had shiny crystals in it. The differential diagnosis was DC as the most probable diagnosis then unicystic ameloblastoma. The patient received antibiotic therapy to avoid secondary infection (amoxicillin/ clavulanic acid (Augmentin®, GlaxoSmithKline, London, UK) 625 mg Per-oral (P.O.) every 8 hours for five days).

The findings were explained to the patient regarding the presence of a large cystic lesion, impacted lower 3rd molar with proximity to the nerve canal, weakness of the lower jaw, and external resorption of the lower 2nd molar distal root. Complete removal of the lesion with tooth extraction was not recommended because of the high risk of inferior alveolar nerve trauma and mandibular fracture. Marsupialization was chosen because it is a simple procedure that can reduce the cyst's size over time. The steps were explained briefly to the patient including creating a window in the cystic cavity, removal of a portion of the lining for biopsy, and packing the cavity with gauze. The patient was informed about the need for multiple follow-up visits. Periodic X-ray films will be taken to check the movement of the 3rd molar, and orthodontic extrusion or surgical extraction will be necessary later. The patient agreed to the treatment plan and signed written consent, but he did not show up for the next appointment.

The patient disappeared for six months and returned to request surgical treatment. A new panoramic radiograph was taken, which revealed cystic cavity shrinkage and bone cortex remodeling as a result of the needle aspiration decompression. Moreover, the third molar tilted distally a few millimeters away from the 2nd molar (Figure 1b).

Surgical Procedure

The patient was rinsed with 0.12% chlorhexidine gluconate oral rinse PerioGard (Colgate-Palmolive, Salford, United Kingdom) 5 minutes preoperatively. The procedure was done under local anesthesia by inferior alveolar nerve block and buccal infiltration with 2% lidocaine and 1:80,000 epinephrine. One centimeter rounded incision was done posterior to the lower 2nd molar then the muco-periosteal flap was removed (Figure 1c). Drilling with a straight surgical handpiece and a fissure bur (Carbide Bur 2.35mm HP 559, Wave Dental, China) was performed to create a bony window. The roof of the cystic lining under the formed window was excised and taken as an incisional biopsy besides the removed bone cortex. The cyst fluid was then evacuated, and the cavity was irrigated with normal saline which was free of any mass. The cystic lining was sutured to the surrounding oral mucosa with 4-0 polyglycolic acid (PGA RESORBA, RESORBA Medical GmbH, Nurnberg, Germany). Gauze soaked in Fusidic acid was used to pack the cystic cavity (Fucidin cream 2 %, LEO Pharmaceutical Products, Ballerup, Denmark). A suture was done across the pack to fix it in place.

The postoperative medications included amoxicillin/clavulanic acid (Augmentin®) 625 mg P.O. every 8 hours for five days, ibuprofen (Brufen, Hamol Limited, Nottingham, England) 400 mg P.O. every 8 hours for three days, and 0.12% chlorhexidine mouthwash (3M™, Peridex™, 3M, St. Paul, Minnesota) every 12 hours for seven days. The patient was instructed to have a strict soft food diet to avoid jaw fracture due to bone weakness.

Follow-up

The wound healing was adequate two weeks after surgery. The gauze was removed, the cavity was irrigated with normal saline, and a new smaller pack soaked in Fucidin cream was placed. During the next two weeks, the patient was requested to come in every three days to change the gauze pack to a smaller one, then every seven days for the next three weeks. The pack was then removed, and the patient was given a syringe to irrigate the cavity twice daily with regular saline.

Biopsy Result

**Figure 2:**
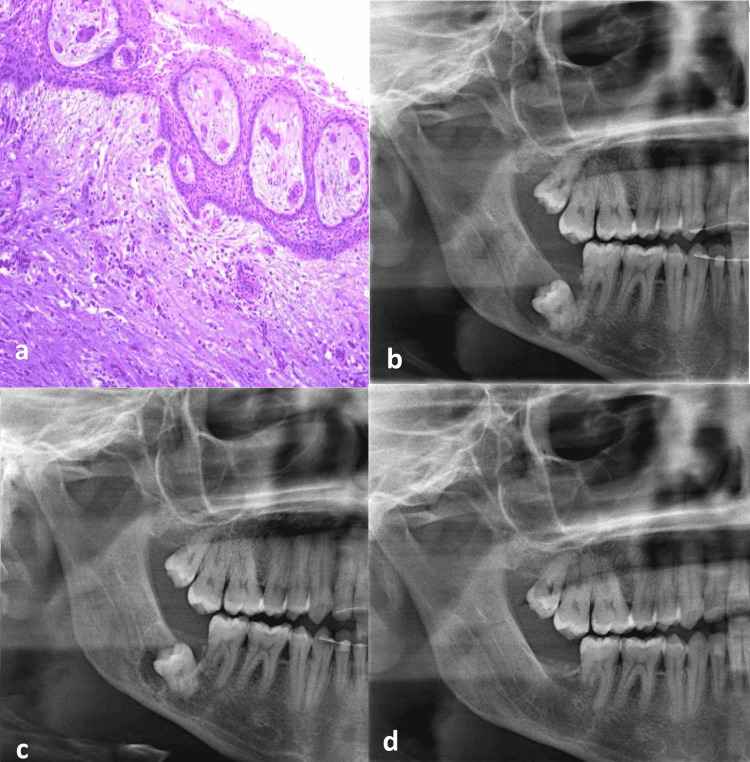
Biopsy result and follow-up images (a) The histopathology section (b) 4 months after marsupialization (c) 8 months after marsupialization: more shrinkage of the cyst (d) 15 months after extraction: complete bone healing was achieved.

The histopathology section reveals a cystic lesion of fibrous connective tissue wall with the focal lining of hyperplastic non-keratinized epithelium showing elongated interconnecting rete ridges. There is focal chronic inflammatory cellular infiltrate and foci of odontogenic epithelial rests with small inactive appearing. Based on the biopsy result, the lesion was diagnosed as inflamed DC (Figure 2a). The patient came four months after marsupialization for follow-up (Figure 2b). Although the cystic cavity shrank, it still enclosed the inferior alveolar canal. The decision was made to wait until the cavity's edge was clear of the canal. A panoramic radiograph was taken after additional four months when the patient returned to the country after being abroad for study. It showed a reduction in the distal and inferior borders of the cystic cavity with a limited occlusal and distal movement of the tooth. Moreover, the bone on the lower border of the mandible became thicker with adequate new bone formation allowing the extraction of the third molar with minimal risk of bone fracture (Figure 2c). Complete bone healing was achieved after 15 months of extraction (Figure 2d).

Surgical Extraction

Under local anesthesia, a mucoperiosteal flap was reflected, and bone reduction was used to expose the affected third molar utilizing a surgical straight handpiece and round bur. The roots were separated by fissure bur, then the roots were luxated and extracted non-traumatically. The window was closed primarily by suture. The same drugs were used for post-operative therapy as for the marsupialization surgery. During the follow-up, the patient's lower lip and skin had normal sensations. The most recent panoramic radiograph was 15 months after the surgical extraction, and it revealed complete bone healing at the extraction site. The second molar's distal root was partly resorbed, but the tooth was clinically asymptomatic and had a positive response to the cold pulp test. Based on the pulp assessment there was no need for root canal therapy (Figure 2d).

Case 2

In January 2019, a 36-year-old healthy male patient requested dental care at the University clinics. A panoramic radiograph was taken as a part of the ordinary radiographic evaluation. An impacted third molar was accidentally detected with radiolucency around the crown.

Clinical Examination

Extra-oral examination showed a symmetric face with normal skin. Intra-orally, the lower left third molar was missed with normal retromolar mucosa.

Radiographic Examination

**Figure 3:**
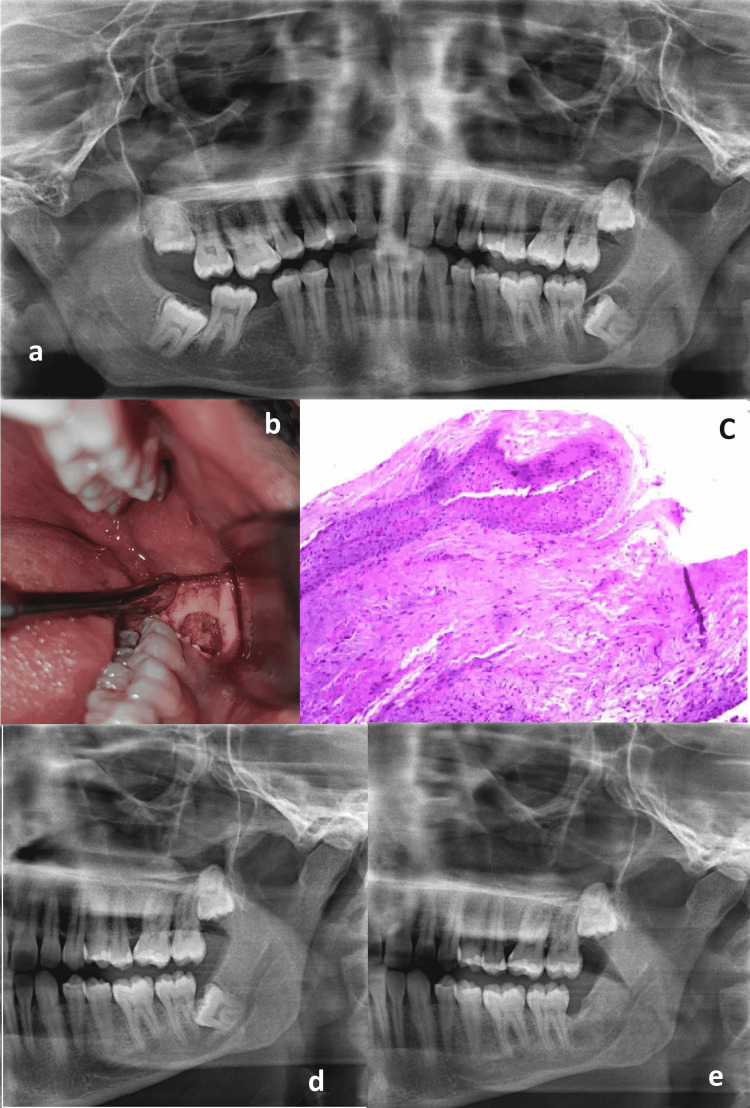
Preliminary diagnosis, surgical procedure, biopsy results, follow-up schedule to achieve complete bone healing (a) Initial panoramic radiograph presented a cystic lesion (b) a window was created as a part of marsupialization (c) The histopathology section (d) 8 months after marsupialization: more shrinkage of the cyst (e) 14 months after extraction: Complete bone healing was achieved

A mesioangular impacted lower left third molar with divergent roots near the inferior alveolar canal was seen. A well-defined radiolucent lesion with a radio-opaque border surrounded the crown. This lesion extended distal to the crown, mesially toward the apex of the lower second molar's mesial root, and downward toward the inferior alveolar canal (Figure 3a). DC was the preliminary diagnosis based on the radiographic and clinical findings. Enucleation was ruled out as a treatment option since the lesion's border extended beyond the inferior alveolar canal, putting the nerve at risk during cystic lining removal. Marsupialization was indicated to shift the cyst lesion away from the canal. The third molar tooth can be orthodontically extruded or extracted later. Figure 3 summarizes the biopsy results, surgical procedure, and follow-up schedule to achieve complete bone healing. 

Surgical Procedure

Aspiration of the cystic fluid using the same method in case 1 revealed a clear pale straw color. Marsupialization was accomplished using the same surgical methods as in case 1, including the creation of a window, an incisional biopsy of the cystic lining beneath the window, aspiration of cystic fluid, packing, and suture (Figure 3b). The same follow-up protocol was used as well.

Biopsy Result

A fibrous connective tissue wall with a lining of the hyperplastic non-keratinizing stratified squamous epithelium was seen in a histopathologic section. There are no signs of inflammation. The final diagnosis was a non-inflamed DC (Figure 3c).

Surgical Extraction

A panoramic radiograph was taken four months after marsupialization and revealed that the cyst had shrunk, but that there was still a radiolucency mesial to the third molar and apical to the second molar. As a result, the surgical extraction has been postponed. The patient came after additional four months and a panoramic radiograph was conducted, the cystic lesion distal to the third molar and apical to the 2nd molar had entirely resolved. However, there was only a small radiolucency anterior to the mesial wall of the 3rd molar crown and posterior to the distal root of the second molar (Figure 3d). Enucleation of the lining and surgical extraction were performed under local anesthesia using the same technique used in case 1. After the operation, the lower lip and skin had a normal sensation. The last follow-up visit was after fourteen months and a new panoramic radiograph demonstrated complete bone repair (Figure 3e).

## Discussion

DC presents in two different forms: developmental and inflammatory. The asymptomatic developmental cyst affects adult teeth in the late second or third decades of life. On the other hand, pulp infection on the ancestor primary tooth stimulates the production of the inflammatory type, which involves the follicle of immature permanent teeth. The first and early part of the second decade are the most common times for this type to be diagnosed [1]. The two DCs described in this report were considered to be developmental because both cysts affected mature lower third molars in the patients, who were 25 and 36 years old, and the cyst formation was not initiated by infection. The cyst in the first case, on the other hand, is inflamed since it was aspirated a few months before the biopsy. The second patient came to the clinic for routine dental treatment with no swelling or other symptoms, and the DC was discovered using a panoramic radiograph. The missing tooth from the dental arch, particularly the third molars, may be impacted or involved by a lesion that impedes eruption such as DC.

Treatment of DCs associated with third molars varies from similar cysts that involve other teeth, as saving third molars in the dental arch is not necessary. Furthermore, the position of the third molar is much closer to the inferior alveolar canal than other teeth especially if the tooth has been displaced inferiorly or posteriorly by the cyst. Enucleation of the cystic lining and extraction of such teeth puts the inferior alveolar nerve in serious danger, as well as causing further mandibular bone weakening and risk of pathologic fracture. The recommended therapy is marsupialization, with the goal of moving the cystic cavity and the implicated third molar away from the canal before attempting to remove the cyst lining. The tooth in concern may erupt on its own, be extruded by orthodontic treatment, or be surgically removed [8].

It is worth noting that aspiration of part of the cystic fluid by the needle was capable of producing adequate decompression that resulted in significant shrinkage of the cystic cavity and bone deposition within 5-6 months. This result supports that decompression is an essential factor for bone deposition and reducing the size of cystic lesions [9]. The marsupialization procedures were carried out simply by establishing a window, with no tube or other device used to keep the window open. The two patients were able to clean the cystic cavity by rinsing with Chlorohexidine mouthwash within the two post-operative weeks, then by daily mouthwash without any incidence of infection.

The studies that evaluated the indicators for a spontaneous eruption and a cyst-associated tooth were systematically reviewed by Nahajowski et al. [10] who concluded that the most critical factors are the patient's age younger than 10 years, less than half root formation. Other factors are tooth depth, inclination, and appropriate space [11]. If the involved tooth is unable to erupt, it can be forcibly extruded by orthodontic tools and aligned within occlusion. Abu-Mostafa and Abbasi [12] published a case report in 2017 in which they effectively marsupialized a large DC and extruded the three involved permanent teeth in a 12-year-old female patient. In the year 2020, Chung et al. [13] successfully treated a 10-year-old boy who had a large DC on the mandible and unerupted first and second permanent molars. The cystic lesion was marsupialized with orthodontic traction of the two molars using an orthodontic miniplate anchorage between the mandibular premolars. Without the use of a full fixed device, the movement was directed by an orthodontic miniplate on the opposing arch. Full eruption of the two molars was achieved after four years of treatment. In the current report, both cyst-associated third molars were unable to erupt spontaneously, however, the tooth in the first case moved a few millimeters distally. In the second case, the third molar was mesioangular angulated and the way of the eruption was impeded by the adjacent second molar.

The recommended time for enucleation of the remaining cystic lining after marsupialization depends on the size of the cyst and the relation with adjacent vital structures. In 2021, Irimia et al. [14] marsupialized a large residual cyst on the mandible that extended from the 1st premolar area to the coronoid process. Decompression was achieved by cutting a window in the alveolar process, with a six-month follow-up period. The cystic cavity shrank dramatically, allowing for additional surgery for enucleation of the residual lining. They guided bone healing with a titanium mesh, and the result was proper bone reconstruction. Surgical removal of the cystic linings and the involved teeth were performed in the current case reports after eight months. Due to the prolonged follow-up period, bone deposition and shrinkage of the very large cystic cavity in case 1 of the present report was possible. Moreover, the patient's cooperation for recall visits and oral hygiene were essential rules for successful treatment outcomes. 

The initial treatment plan for the present two cysts was marsupialization followed by extrusion of the cyst-associated teeth by orthodontic devices. Then, the teeth can be extracted after being away from the inferior alveolar canal. A similar technique was utilized successfully by Montevecchi et al. in 2012 [8]. However, the two patients in this report were unable to receive orthodontic treatment because of being studying abroad or budgetary constraints. As a result, when the cystic lesions reduced in size and relocated somewhat away from the canal, the linings were removed and surgical extraction was performed. A low-speed surgical handpiece with a fissure bur was used to create a gap around the crown, and subsequently, root separation was used to retrieve both cyst-associated teeth. The extraction procedures were atraumatic and neither patient's lower lip had any neurosensory alteration as result. The softness of the newly created bone around the teeth may have allowed the divided roots to be luxated and removed without causing hemorrhage or sensory disturbance of the inferior alveolar nerves.

## Conclusions

The needed decompression for bone deposition and reduction of large DCs can be achieved with simple marsupialization by establishing a window. Treatment success is dependent on an extended period of follow-up and the patient's motivation to maintain proper oral hygiene. Surgical extraction can be performed atraumatically by minimal bone reduction, root separation, and cautious luxation of the roots of the cyst-associated tooth because the surrounding bone is newer, less calcified, and softer than mature bone. A panoramic radiograph is recommended as a part of ordinary radiographic examination, especially for patients who have an incomplete eruption of the full dental arch.
